# Insights into “Yin Rhyme”: Analysis of nonvolatile components in Tieguanyin oolong tea during the manufacturing process

**DOI:** 10.1016/j.fochx.2024.101729

**Published:** 2024-08-12

**Authors:** Qiuming Li, Qingcai Hu, Xiaoxi Ou, Jihang He, Xinru Yu, Yunzhi Hao, Yucheng Zheng, Yun Sun

**Affiliations:** a Key Laboratory of Tea Science, College of Horticulture, Fujian Agriculture and Forestry University, Fuzhou 350002, China; b College of Tea and Food Sciences, Wuyi University, Tea Engineering Research Center of Fujian Higher Education, Tea Science Research Institute of Wuyi University, Wuyishan 354300, China

**Keywords:** *Camellia sinensis*, Oolong tea, “Yin Rhyme”, Manufacturing process

## Abstract

Tieguanyin (TGY) is renowned for its distinctive “Yin Rhyme” flavor. To elucidate the underlying formation mechanism, we conducted sensory evaluations, electronic tongue analysis, and widely-targeted metabolomics. Our sensory evaluations and electronic tongue results indicated that TGY exhibits a thick and mellow taste profile, contributing to the “Yin Rhyme” flavor. Metabolomics analysis of tea products revealed that TGY shows significantly higher concentrations of umami substances (L-glutamate, L-theanine) and bitter substances (valine, catechins) compared to Jinguanyin (JGY). Additionally, metabolomic analysis during different oolong tea processing stages revealed significant differences in 21 substances, including L-glutamate, L-theanine, valine, and catechins, between fresh leaves of both varieties. These substances exhibited distinct fluctuation patterns during processing, indicating that the cultivar plays a crucial role in developing the “Yin Rhyme” flavor, which was enhanced throughout processing. This study provides a theoretical foundation for understanding the formation of the unique “Yin Rhyme” flavor of TGY.

## Highlights

• Tieguanyin’ s thicker, mellower taste profile leads its unique ‘Yin Rhyme’ flavor.
• Higher levels of L-glutamate and L-theanine contribute to the ‘Yin Rhyme’ flavor.
• Post-harvest processing of oolong tea is crucial for ‘Yin Rhyme’ flavor development.
• Special compounds in fresh leaves of Tieguanyin is key to ‘Yin Rhyme’ development.

## 1 Introduction

Oolong tea stands out among its counterparts due to its inherent floral and fruity aroma, in addition to its smooth and invigorating taste profile. Consumers are drawn to it not only for its diversity but also for its intricate and distinct processing methods (Zeng et al., 2020). The distinctive flavor of oolong tea is intricately linked to its processing techniques and superior cultivars. The primary constituents influencing its taste are nonvolatile compounds, which develop and accumulate through enzymatic processes (e.g., withering, rolling, and oxidation) and nonenzymatic processes (e.g., firing) (Wu et al., 2020). The abundant metabolites in oolong tea, such as amino acids, flavonoids, alkaloids, and sugars, collectively contribute to flavor characteristics.

Fujian Province, the location where oolong tea originated, has abundant oolong tea germplasm resources. ‘Jinguanyin’ and ‘Tieguanyin’ are representative varieties of oolong tea, and their production and applications contribute to enriching the quality characteristics of existing tea leaves and the importance of tea in the local economy. *Camellia sinensis cv. Tieguanyin* (TGY, no. GS13007) is the highest grade of oolong tea due to its unique “Yin Rhyme” flavor, which can be described as a mellow and thick taste, and its freshness (Cao et al., 2022; Li et al., 2023). Guo et al. (2019) suggested that specific secondary metabolites may contribute to the development of the “Yin Rhyme” flavor in TGY. The taste characteristics of *Camellia sinensis cv. Jinguanyin* (JGY, no. GS2002017) are very similar to those of TGY, with a mellow and sweet aftertaste (Hu et al., 2023), but it lacks the “Yin Rhyme” flavor. Hence, “Yin Rhyme” is the typical taste characteristic of the TGY cultivar.

Comprehensive insights into taste formation patterns and the underlying mechanisms during oolong tea production have been obtained (Chen et al., 2020; Wu et al., 2020). Recent research has increasingly illuminated the impact of the specific production stages on oolong tea taste, including withering (Huang et al., 2020; Jia et al., 2023; Wang et al., 2019), the turnover phase (Huang et al., 2020), and the firing processing (Cao et al., 2021; Peng et al., 2022). Moreover, studies have indicated that external stresses during processing facilitate the accumulation of essential taste compounds, such as soluble sugars (Scharbert & Hofmann, 2005; Troszyńska et al., 2010), alkaloids (Kim et al., 2011), amino acids (Chen et al., 2020; Feng et al., 2014; Ren et al., 2021; Yu & Yang, 2020), catechins (Thorngate & Noble, 1995), and flavonol glycosides (Dou et al., 2007; Fang et al., 2020), in oolong tea. These stresses synergistically interact to promote the development of the taste profile of oolong tea. Additionally, previous studies have validated the flavor of each component through a default experiment after recombination experiments (Kaneko et al., 2006; Scharbert & Hofmann, 2005; Yu et al., 2014), influencing the comprehensive taste characteristics of tea through the coordination of types, content, and proportions. TGY is a characteristic variety of oolong tea, and its “Yin Rhyme” flavor is the key factor determining the flavor of the tea product, which also has a direct connection with market prices. However, current research in this area has focused only on the sensory quality of tea and the targeted biochemical components of the tea products (Xin et al., 2012) and has not comprehensively identified the main substances that form the “Yin Rhyme” flavor. At the same time, the “Yin Rhyme” flavor of TGY often appears in tea products, but Guo et al. (2019) examined only fresh leaves and failed to provide an accurate description of the “Yin Rhyme” flavor of TGY. There is still no scientific or systematic theoretical explanation for the mechanism of formation of the unique “Yin Rhyme” flavor of TGY. Hence, we conducted comparative analyses using TGY as the subject and JGY as the reference. At the same time, we scrutinized the flavor attributes and nonvolatile taste components of JGY and TGY tea products, as well as the changes that occur during processing.

Initially, sensory evaluation and electronic tongue technology were integrated in this study to preliminarily identify differences in taste between the two oolong tea varieties (TGY and JGY). Next, a comparative analysis of the nonvolatile metabolites in the tea products was carried out, further confirming the “Yin Rhyme” flavor compounds of TGY. Ultimately, by examining the dynamic alterations in the nonvolatile flavor constituents of both TGY and JGY throughout the processing stages, systematic insights were gained into the evolution of the unique “Yin Rhyme” flavor in TGY.

## 2 Materials and methods

### 2.1 Tea sample preparation and extraction

This experiment used the elite tea cultivars Tieguanyin (*Camellia sinensis cv.* Tieguanyin, TGY) and Jinguanyin (*Camellia sinensis cv.* Jinguanyin, JGY) to produce oolong tea samples. Fresh leaves were harvested from the ecological tea garden of Juyuan village, Longjuan township, Anxi County, Quanzhou, Fujian (24.55°N, 117.50°E), with the standard being a healthy, pest-free single bud with three to four leaves.

**Fig. 1:**
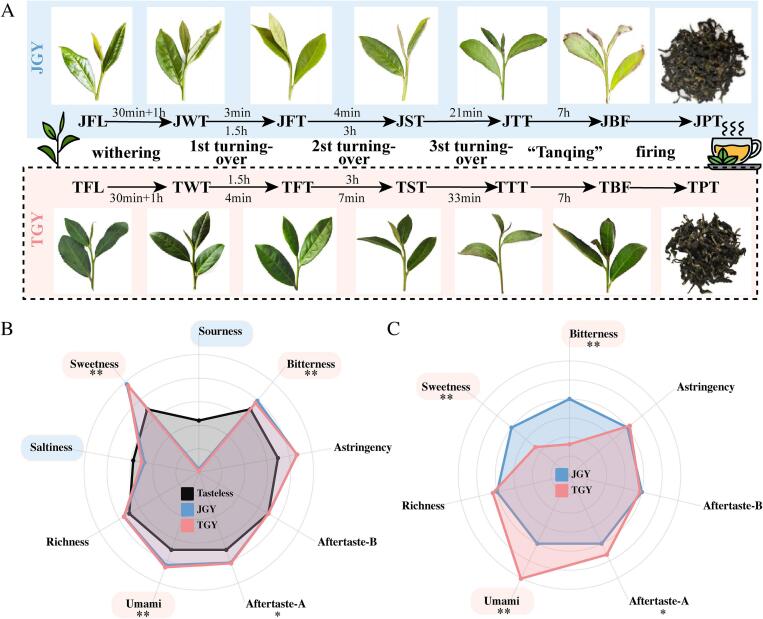
(A) Simplified flowchart of TGY and JGY manufacturing, sample information and working times of various processes during the processing of oolong tea. JGY: Jinguanyin; TGY: Tieguanyin; TFL: TGY fresh leaf; TWT: TGY after withering; TFT: TGY after the 1st turnover; TST: TGY after the 2nd turnover; TTT: TGY after the 3rd turnover; TBF: TGY before firing; TPT: TGY tea product; JFL: JGY fresh leaf; JWT: JGY after withering; JFT: JGY after the 1st turnover; JST: JGY after the 2nd turnover; JTT: JGY after the 3rd turnover; JBF: JGY before firing; JPT: JGY tea product. (B—C) Sensory quality characteristics of TGY and JGY finished teas determined by electronic tongue (*E*-tongue) analysis. (B) Values of various taste attributes compared with a tasteless solution. (C) Values of various taste attributes for TGY and JGY. Tasteless: reference solution; ** represents a significant difference of *p* < 0.01; * indicates a significant difference of *p* < 0.05.

First, to minimize the impact of other experimental factors, both JGY and TGY fresh leaves that met the sane standards were harvested from identical locations at nearly simultaneous harvesting times. Specifically, fresh leaves of JGY (JFL) were collected on April 25, 2021, and fresh leaves of TGY (TFL) were collected on April 27, 2021. Furthermore, to mitigate the impact of processing techniques on tea quality, the processing methods (Wu et al., 2020) for both TGY and JGY were kept relatively consistent (Fig. 1A), with three controlled turnover treatments. The fresh leaves of both plants were processed at the same location, with the processing parameters and sample collection points shown in Fig. 1A.

The fresh leaves were subjected to 30 min of sun withering followed by 1 h in an indoor setting, which resulted in withered leaves (TWT, JWT). After the first turnover treatment of JGY and TGY for 3 min and 4 min, respectively, followed by 1.5 h in an indoor setting, we obtained the first turnover leaves (TFT, JFT). The second turnover treatment was performed for 4 min and 7 min, respectively, followed by 3 h in an indoor setting to obtain the second turnover leaves (TST, JST). After the third turnover treatment for 21 min and 33 min, respectively, we obtained the third turnover leaves (TTT, JTT). Before firing, we obtained leaves (TBF, JBF) with a long 7-h “Tanqing”. Each setting step was conducted in an air-conditioned room with a consistent rotation speed of 22 r/min, a temperature of 20 °C and a relative humidity of 60%. Finally, the firing machine temperature was set at 270 °C for a 3 min firing process. Then, the leaves (TBF, JBF) were rolled and dried at 65 °C to produce the tea products (TPT, JPT). Each tea sample was comprised of three separate biological replicates.

Finished tea samples of JGY and TGY (TPT, JPT) were used for electronic tongue and sensory analysis (Fig. 1A), and seven key process sample leaves were used for nonvolatile metabolite analysis. Every tea sample was instantly frozen in liquid nitrogen and stored at −80 °C.

### 2.2 Sensorial analysis

Following prior research (Xu et al., 2022; Zheng et al., 2022), sensory assessments of JGY and TGY tea products were carried out by a group comprised of 5 female and male members between 20 and 50 years of age who all had more than 5 years of experience in sensory analysis and had undergone training. The brewing procedure followed the 1:50 tea-to-water ratio cylindrical cup method outlined in GB/T 23776–2018 of immersing 3.0 g of the tea products in boiling water for 5 min. Subsequently, we recorded and scored the intensity (0−10) of the tasting attributes (richness, freshness, astringency, bitterness, and thickness), similar to methods outlined by Guo et al. (2021). The tea samples were accessed in accordance with GB/T 23776–2018, and ethical permission was not required. The appropriate protocols for protecting the rights and privacy of all participants were utilized during the execution of this research. All panelists were informed and the informed consent was obtained from all participants for this experiment.

### 2.3 The electronic tongue (*E*-tongue) analysis

An electronic tongue (TS-5000Z, Insent Electricity Company, Japan) with six fundamental taste sensors was used to evaluate the taste attributes of the tea product, facilitating the assessment of diverse taste properties. In brief, tea products of JGY (3.0 g) and TGY (3.0 g) were brewed with 150 mL of boiling water for 5 min. Then, the mixture was quickly filtered and cooled to room temperature. After that, three biological replicates of the filtered tea liquid were used as an independent sample. At the same time, the reference and pretreatment solutions for the taste sensors (a flavorless solution) were composed of a mixed 0.3 mM KCl and tartaric acid solution. The point of tastelessness for sourness was −13, and that for saltiness was −6. When the taste value of a sample was below the point of tastelessness, it indicated that the sample did not have flavor and vice versa.

### 2.4 Widely-targeted metabolomic analysis of nonvolatile compounds in TGY and JGY tea samples

Nonvolatile compounds were analyzed by MetWare Biotechnology Co., Ltd. (Wuhan, China) according to methods outlined by Wu et al. (2020). In brief, for the preparation of samples for detection, all the samples were freeze-dried using a vacuum freeze dryer (Scientz-100F). Then, all the samples were ground into powder using a mixer mill with zirconia beads (MM400, Retsch) at 30 Hz for 1.5 min. We dissolved 100 mg of powder in 1.2 mL of a 70% methanol solution. The material was swirled 6 times, each time for 30 s at 30 min intervals. Then, the samples were placed overnight at 4 °C. Next, the samples were centrifuged at 12,000 rpm for 10 min, after which the supernatant was collected. Finally, we filtered the sample (SCAA-104, pore size 0.22 μm) and performed metabolomics analysis on the UPLC-ESI-MS/MS system.

The parameters of the UPLC column (Agilent SB-C18, 1.8 μm, 2.1 mm × 100 mm) were as follows: mobile phases A and B were pure water containing 0.1% formic acid and acetonitrile containing 0.1% formic acid, respectively. The flow rate was 0.35 mL/min, the column temperature was 40 °C, and the injection volume was 4 μL. A gradient program was used to obtain sample measurements, starting with 95% A and 5% B, which were linearly programmed to 5% A and 95% B within 9 min and maintained for 1 min. Subsequently, the program returned to the initial conditions within 11 min, and the initial conditions were maintained for 14 min.

The ESI source operating parameters were as follows: ion source, turbo spray, source temperature 550 °C, and ion spray voltage (IS) 5500 V (positive ion mode)/−4500 V (negative ion mode). The ion source gas I (GSI), gas II (GSII), and curtain gas (CUR) were set at 50, 60, and 25.0 psi, respectively, with high collision-induced dissociation parameters. A triple quadrupole linear ion trap mass spectrometer (Q TRAP), the AB4500 Q TRAP UPLC/MS/MS system, equipped with an ESI Turbo ion spray interface to control positive and negative ion operation modes, was used to obtain LIT and triple quadrupole (QQQ) scans. During QQQ and LWT mode operation, we tuned and calibrated the instrument using 10 and 100 μmol/L polyethylene glycol solutions. Then, we monitored a specific group of MRM ion pairs based on the eluted metabolites in each period.

Metabolite qualitative analysis was performed based on retention time (RT), fragment pattern, accurate *m*/*z* values, and a self-designed database (MWBD, Metware, Wuhan, China) (Chen et al., 2013). Quantitative analysis was conducted using multiple reaction monitoring (MRM) based on triple quadrupole mass spectrometry. After obtaining the mass spectrometry analysis data of different samples, we integrated the peak areas of all substance mass spectrometry peaks and performed peak area integration correction for the mass spectrometry peaks of the same metabolite in different samples (Fraga et al., 2010).

### 2.5 Data analysis

The data were analyzed as the average of three biological replicates. T-tests were performed on the electronic tongue data to assess statistical significance and determine if there were differences between the two varieties. Additionally, various statistical analysis methods based on those in previous studies were applied to the metabolite data (Pang et al., 2023; Thévenot et al., 2015). We used R version 3.5.1 software with default parameters for principal component analysis (PCA) and orthogonal partial least squares discriminant analysis (OPLS-DA). Hierarchical clustering analysis (HCA) was conducted using TBtools. In addition, significant differences in metabolites between TGY and JGY under the same processing conditions were identified by using t-tests, OPLS-DA and fold change (FC) criteria. The criteria for selecting significantly upregulated different compounds were VIP ≥ 1 and FC ≥ 2, while the criteria for selecting significantly downregulated different compounds were VIP ≥1 and FC ≤ 0.5. Finally, Graphpad Prism 9.0 was used to create bar graphs and line graphs, and Adobe Illustrator 2023 was employed for graphic layout. All the data are included in the supplementary dataset.

## 3 Results and discussion

### 3.1 A comparison of the taste characteristics of TGY and JGY tea products

#### 3.1.1 Results of the sensory evaluation of the tea products

To analyze the taste characteristics of TGY tea products, we compared them with those of JGY tea products for sensory evaluation. As shown in Table 1, both JGY and TGY had mellow and refreshing taste characteristics. However, the taste of TGY was richer and mellower, and the tea soup was thicker and fresher. In particular, it had a “Yin Rhyme” flavor. In contrast, JGY had a relatively sweeter aftertaste. Hence, “Yin Rhyme” was the typical taste characteristic of the TGY cultivar.

**Table 1:** Sensory quality of tea products of TGY and JGY.

Tea Sample	Liquor color		Aroma		Taste	
	Comment	Score	Comment	Score	Comment	Score
TPT	Light yellow, bright	93.12 ± 0.63	High and clean flower aroma	95.04 ± 0.35	Thickness, mellow and fresh, “Yin Rhyme”	92.1 ± 0.26
JPT	Golden yellow, bright	87.98 ± 0.75	Sweet flower and fruit aroma	92.88 ± 0.33	Mellow and fresh, sweet in aftertaste	89.94 ± 0.34

Note: Data are presented as mean ± standard deviation (*n* = 5); TPT: TGY tea product; JPT: JGY tea product; Each attribute was evaluated on a scale of 100.

#### 3.1.2 Results of the electronic tongue (*E*-tongue) evaluation of the tea products

The E-tongue can be used to forecast sensory features and their relationship to the taste quality of tea as assessed by a professional taster (He et al., 2009). To minimize the influence of subjective factors in sensory evaluation on taste outcomes, we further analyzed the taste characteristics of TGY tea products by conducting electronic tongue tests. The results indicated that a total of eight taste indicators were obtained, with the sourness and saltiness of both teas found to be below the tasteless threshold (Fig. 1B). Therefore, these two taste indicators were excluded from this study. Furthermore, to highlight the differences in taste indicators between JGY and TGY, we used JGY as the reference point for comparison (Fig. 1C). Previous studies have shown that the “Yin Rhyme” flavor is related to the accumulation of high levels of catechins, caffeine, and limonene (Guo et al., 2019). The electronic tongue results showed that these flavor sensations were more pronounced in the TGY than in the other sample and were related to the formation of the “Yin Rhyme” flavor (Fig. 1C). Specifically, the umami value of TGY was significantly greater than that of JGY, and the astringency aftertaste value was also greater than that of JGY. However, the bitterness and sweetness of this cultivar were markedly lower than those of JGY. The difference in richness was not significant, but the TGY was still slightly greater than the JGY. In summary, we speculated that the “Yin Rhyme” of TGY is related to the cooperative effect of rich, sweet, bitter, and astringency-aftertaste substances, and thus we conducted metabolomics research to clarify the mechanism of its formation.

#### 3.1.3 Identification of key metabolites involved in “Yin Rhyme” flavor formation in TGY

Nonvolatile metabolites are key factors in the formation of tea flavor characteristics. To better understand the changes in the nonvolatile metabolites of the tea products of TGY and JGY, the samples were tested using comprehensive and widely-targeted metabolomic techniques. A total of 1317 metabolites were detected, including 98 amino acids and derivatives, 242 phenolic acid compounds, 74 nucleotides and derivatives, 324 flavonoids, 94 alkaloids, 95 organic acids, 145 lipids, 29 terpenoids, 47 lignans and coumarins, 78 saccharides and alcohols, 31 tannins and 60 others. Specifically, 1095 compounds were detected in TGY, and 1005 were detected in JGY. In addition, 783 compounds were common to both, with 312 compounds unique to TGY and 222 compounds unique to JGY. The diversity of these substances establishes the basis for the differences in their flavor quality.

**Fig. 2:**
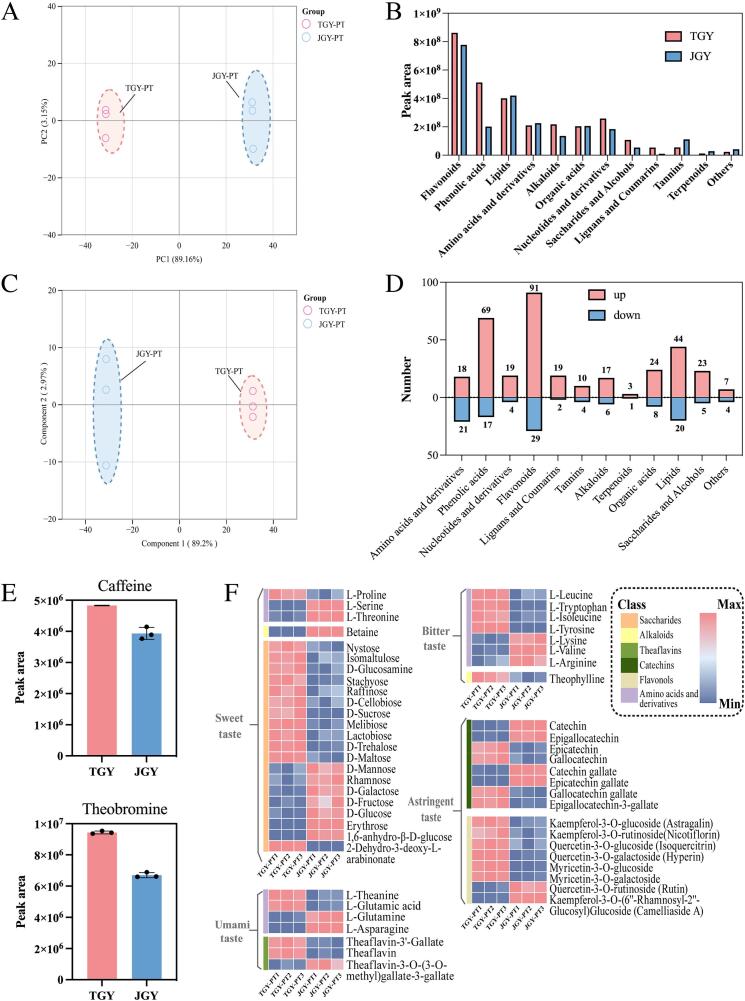
Profiles of nonvolatile metabolites from TGY and JGY oolong tea samples in tea products. (A) PCA score plot of TGY and JGY tea products based on the metabolomic dataset. (B) Comparison of the distribution of all the nonvolatile categories in the two varieties. (C) The OPLS-DA score plot of TGY and JGY tea products based on the metabolomic dataset. TGY-PT: TGY tea product; JGY-PT: JGY tea product. (D) The number of significantly differentially abundant metabolites in various classes with the criteria of fold change ≥ 2 or ≤ 0.5, VIP values ≥1.0 and *p* values < 0.05. The numbers labeled on each class represent the amounts of up- or downregulated metabolites. up: upregulated metabolites; down: downregulated metabolites. (E) Nondifferential metabolites in the tea products of JGY and TGY. (F) Heatmap of characteristic metabolites from TGY and JGY oolong tea samples.

Subsequently, we conducted a comparative analysis of the total peak areas of 12 compound categories between the two cultivars. As illustrated in Fig. 2B, the predominant proportions of peak areas in both varieties were attributed to flavonoids, phenolic acids, and lipids. Specifically, the peak areas of flavonoids, phenolic acids, alkaloids, nucleotides and their derivatives in TGY surpassed those in JGY. Notably, the peak areas of saccharides and alcohols in TGY were markedly greater than those in JGY. Nevertheless, in terms of sensory assessment and electronic tongue data, the sweetness of JGY was significantly greater than that of TGY. This finding implied that the sweetness of tea is influenced by the collaborative interaction of diverse compounds (Troszyńska et al., 2010).

At the same time, the PCA results showed that PC 1 and PC 2 accounted for 89.16% and 3.15%, respectively, of the total variance, and their sum exceeded 90%. This indicated that the experimental data had high accuracy and reliability (Fig. 2A). PC1 showed a distinct separation of tea products of TGY and JGY, with three replicates of the same sample clustering closely together (Fig. 2A). This suggested that there are differences between TGY and JGY in the taste substances of their tea products.

Based on the significant differences in OPLS-DA between TGY and JGY (Fig. 2C), we screened differentially expressed metabolites (DEMs) with restrictions on the *p* value, FC value, and VIP value. Specifically, substances with *p* < 0.05, FC ≥ 2, and VIP ≥ 1 were considered significantly upregulated metabolites, indicating substances that were significantly greater in TGY than in JGY. Substances with *p* < 0.05, FC ≤ 0.5, and VIP ≥ 1 were deemed significantly downregulated metabolites, indicating substances that were significantly lower in TGY than in the JGY (Fig. 2D). A total of 465 common differentially abundant metabolites were screened, among which all categories of compounds in TGY were more abundant than those in JGY. Among them, flavonoids and phenolic acids were the most significant (Fig. 2D). This suggested that the high content of certain secondary metabolites in TGY contributes to the development of its “Yin Rhyme” flavor.

In this study, we analyzed the primary flavor components, including amino acids, catechins, flavonol glycosides, soluble sugars, and alkaloids. The integration of amino acid taste characteristics and their threshold values (Yu & Yang, 2020) revealed notable distinctions in the levels of substances such as L-valine, l-serine, L-threonine, L-glutamic acid, and L-theanine, with relatively lower thresholds and significant content variations serving as key discriminative factors between TGY and JGY. According to the results shown in Fig. 2F, TGY exhibited reduced levels of bitter amino acids such as L-valine and elevated levels of umami amino acids such as L-glutamic acid and L-theanine compared to those in JGY. Notably, the levels of sweet amino acids, including l-serine and L-threonine, were also notably lower in TGY than in JGY. Previous research (Narukawa et al., 2014) has indicated that theanine plays a pivotal role in imparting a mellow and umami taste to tea infusions. Consequently, the abundant theanine content in TGY observed in this study was closely related to its “Yin Rhyme” flavor profile.

**Fig. 3:**
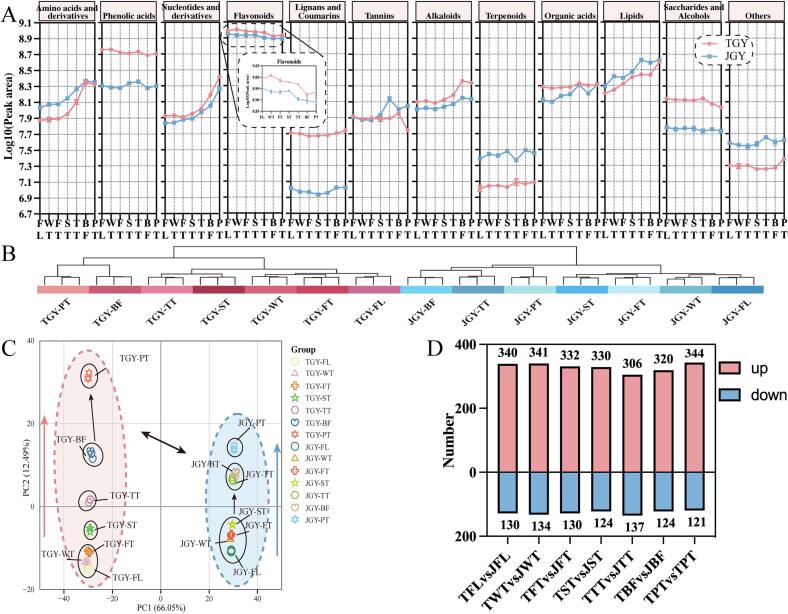
Profiles of nonvolatile metabolites from TGY and JGY oolong tea samples during different production processes. (A) The dynamic trend of various categories of nonvolatile substances during the processing of oolong tea is represented by a line graph. JGY: Jinguanyin; TGY: Tieguanyin; FL: Fresh leaf; WT: After withering; FT: After the 1st turnover; ST: After the 2nd turnover; TT: After the 3rd turnover; BF: Before firing; PT: Tea product. (B) Cluster analysis of different stages of oolong tea processing for the two tea cultivars. (C) The OPLS-DA score plot of TGY and JGY during the manufacturing process. (D) Differentially abundant metabolite numbers with increased and decreased amounts among the comparison groups. up: upregulated metabolites; down: downregulated metabolites.

Catechins play a crucial role in determining the bitterness of tea due to their relatively low threshold values. As shown in Fig. 2F, catechins were the most significantly downregulated substances (15.11 times), and epicatechin gallate was detected only in JGY. Theaflavin-3′-gallate and theaflavin were significantly more abundant in TGY than in JGY by 4.63 and 5.44 times, respectively. This suggested that more tea polyphenols in TGY may be oxidized into flavorful theaflavins. Interestingly, the ester catechins gallocatechin gallate and epigallocatechin-3-gallate were significantly more abundant in TGY than in JGY, which may be the reason for the greater astringency aftertaste of TGY on the *E*-tongue.

Flavonol glycosides are important taste substances with low thresholds and gentle astringency. As shown in Fig. 2F, the levels of kaempferol-3-O-(6″-rhamnosyl-2″-glucosyl) glucoside (camelliaside A) and quercetin-3-O-rutinoside (rutin) were significantly greater in TGY than in JGY, while the levels of quercetin-3-O-galactoside (hyperin), kaempferol-3-O-glucoside (astragalin), myricetin-3-O-galactoside, and myricetin-3-O-glucoside were significantly lower in TGY than in JGY. Specifically, both rutin and hyperin had very low thresholds and showed the largest differences between samples.

Soluble sugars are important sweet substances in tea soup. The results revealed that D-galactose, D-mannose, rhamnose, and d-glucose were the most significantly downregulated substances. Specifically, the galactose content in TGY was 5.71 times greater than that in JGY (Fig. 2F). Moreover, caffeine and theobromine are important bitter substances in tea. Although the difference was not significant between the two clusters, the content was greater in TGY than in JGY (Fig. 2E).

To summarize, the levels of the six sweet compounds, D-galactose, D-mannose, rhamnose, d-glucose, L-threonine, and l-serine, were notably lower in TGY tea than in JGY tea. This disparity serves as the primary distinguishing factor influencing the sweetness contrast between the two types. Subsequently, the presence of valine and catechin was significantly lower in TGY than in JGY, potentially contributing to the distinct differences in bitterness between them. Similarly, the umami-inducing amino acids L-glutamic acid and L-theanine were markedly elevated in TGY, playing a crucial role in the differences in umami taste between the two cultivated varieties. Concurrently, while caffeine content showed no significant divergence between the tea varieties, its formation of a complex with catechin through hydrogen bonding introduces a fresh flavor profile that could further enrich the overall “Yin Rhyme” flavor of TGY. Therefore, the results of the analysis of nonvolatile compounds in the tea products were basically consistent with the results of the electronic tongue and sensory evaluation, which showed that the “Yin Rhyme” flavor of TGY is related to the cooperative effect of rich, sweet, bitter, and astringency-related substances.

### 3.2 Dynamic changes in “Yin Rhyme” flavor formation during the manufacturing process in TGY and JGY

#### 3.2.1 Dynamic changes in nonvolatile components during the manufacturing process

To further investigate the dynamic changes in all detected nonvolatile substances in TGY, a widely-targeted metabolomics analysis was conducted on the 7 consecutive processing steps of TGY and JGY. Fig. 3A shows the dynamic trends of various nonvolatile substances. The results indicated that, in addition to tannins, the contents of the same nonvolatile substances in the two varieties exhibited similar changes during processing (Fig. 3A). Notably, the levels of amino acid derivatives were significantly lower in TGY in the FL and WT stages compared to those of JGY, which started to increase after the first turnover, were particularly pronounced after the second and third turnovers, and peaked in the BF stage, with levels almost equaling those of JGY. This indicates that TGY accumulated more amino acids during the manufacturing process. This may be the result of the accumulation of a large amount of amino acids in TGY in response to stress (Yu & Yang, 2020). JGY showed significantly decreases in flavonoids, which continued to decrease after the ST stage, while TGY showed a significant decrease after TT but did demonstrate some recovery after drying. This may be due to the significant increase in certain flavonoid compounds in TGY under high-temperature conditions. TGY showed an increasing trend in alkaloids during the “Tanqing” process (between the TT and BF stages) that was significantly higher than that of JGY. In summary, during the manufacturing process, TGY accumulated more of these three important flavor substances, which provide the foundation for its “Yin Rhyme” flavor characteristics. Moreover, during the enzymatic oxidation stage of TGY oolong tea processing, the quality formation process was dominated by amino acids and alkaloids, while during the nonenzymatic oxidation stage, the quality formation process was dominated by changes in flavonoid substances.

Subsequently, we further conducted principal component analysis on all samples to visualize the differences among all stages (Fig. 3C). PC 1 was 66.05%, and PC 2 was 12.49%, which accounted for 70% of the total variance, sufficiently representing most of the data variation. The results indicated a significant separation between the two varieties on the first principal component, and each stage exhibited similar distribution patterns during the manufacturing process. This suggested that each stage has key contributors accounting for the differences and that adjacent stages have more similar nonvolatile characteristic substances. Notably, as processing progressed, the greatest difference between the BF and PT stages was observed in the TGY, while in the JGY, the greatest difference was between the ST and TT stages (Fig. 3C). This indicated that during processing, the most significant dynamic changes in nonvolatile substances in the TGY occurred during the firing stage (Fig. 1A). In comparison, the nonvolatile substances in JGY significantly changed between the second and third turnovers. In summary, the dynamic changes in nonvolatile substances at different stages provided the foundation for understanding the differences in taste.

Subsequently, we conducted cluster analysis on all samples to further clarify the differences in each stage (Fig. 3B). The results indicated that due to the differences in the varieties, the nonvolatile substances exhibited significant differences under the same processing conditions. The different varieties can be roughly divided into three stages: early, middle, and late. Among these stages, in the late stages of the TGY, the PT and BF stages formed a branch, and there were significant differences between these stages and the early and middle stages. The results showed that the firing process had the most significant impact on the formation of “Yin Rhyme” flavor in TGY. This may be due to the more intense chemical reactions of certain substances contained in TGY due to the influence of thermal effects.

#### 3.2.2 Dynamic changes in characteristic taste components during the manufacturing process

To further evaluate the common impact of each stage on the metabolite levels of TGY, based on the OPLS-DA setting, the parameters were set to obtain differentially expressed metabolites (DEMs) between the common processing stages of the two varieties (*p* < 0.05, FC ≥ 2 or ≤ 0.5, and VIP ≥ 1). The shared DEMs between the two varieties were considered conservative metabolites induced by specific stages, which better reflect the taste formation pattern during processing. According to the criteria outlined above, the up- and downregulation of the shared DEMs in each process were statistically analyzed (Fig. 3D). The results showed that the number at each stage was relatively close. Notable, the most downregulated substances and the least upregulated substances were in the TT stage. However, the least downregulated substances and the most upregulated substances were in the PT stage. We speculated that the TT and PT stages may be key processing stages for distinguishing between TGY and JGY, which was consistent with the results of the cluster analysis and PCA.

Furthermore, to explore the metabolic pathways of the common DEMs in each stage, KEGG enrichment analysis was conducted. The results indicated that the metabolic pathways were mainly enriched in flavone and flavonol biosynthesis (ko00944), flavonoid biosynthesis (ko00941), galactose metabolism (ko00052), starch and sucrose metabolism (ko00500), and biosynthesis of secondary metabolites (ko01110). The experimental results showed that the different varieties exhibit highly similar conditions at various metabolic levels during the oolong tea manufacturing process.

### 3.3 The metabolic network of characteristic taste components accounts for the dynamic changes between finished and processed tea

The formation of nonvolatile metabolites typically involves multiple pathways, with the transformation network from primary metabolism to secondary metabolism playing a collective role. To explore the formation mechanism of the TGY “Yin Rhyme” characteristic flavor, we compared the dynamic changes in the characteristic flavor substances of the two varieties during the manufacturing process through metabolic pathway analysis. Based on the above analysis and previous studies (Lin et al., 2024), amino acids, flavonoids, soluble sugars, and alkaloids were found to make significant contributions to the flavor characteristics of both. Variations in the content, proportion, threshold, and flavor characteristics of substances led to different taste styles and characteristics of the tea soups.

**Fig. 4:**
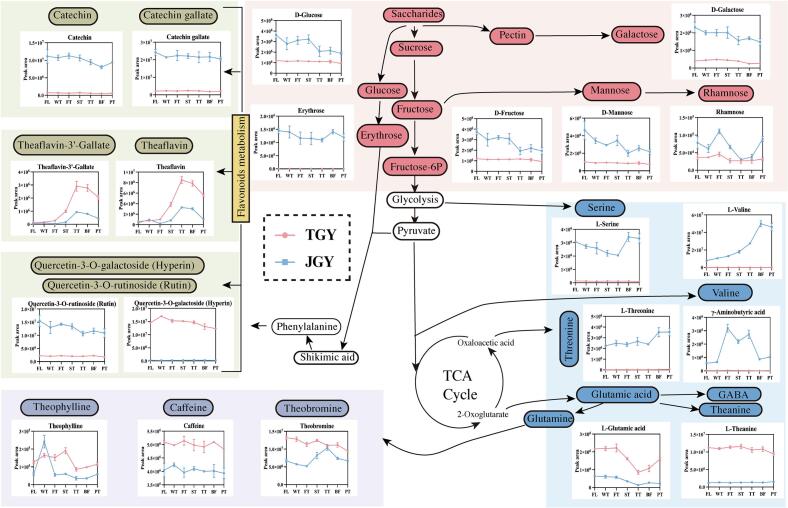
A predictive network map of metabolic pathways associated with flavor formation during the production of Tieguanyin and Jinguanyin oolong teas was constructed by plotting line graphs for each stage. JGY: Jinguanyin; TGY: Tieguanyin; FL: fresh leaf; WT: after withering; FT: after the 1st turnover; ST: after the 2nd turnover; TT: after the 3rd turnover; BF: before firing; PT: tea product.

The quality of tea is significantly influenced by the composition, content, and transformation products of amino acids. Certain amino acids are closely linked to the flavor of tea and serve as essential quality constituents. In Fig. 4, most protein amino acids reached peak levels in the BF stage (L-threonine, L-valine, l-serine, and l-glutamine). Deb and Jolvis Pou (2016) have shown that enzyme hydrolysis of large molecular proteins leads to a significant increase in amino acids. There were significant differences in the accumulation of serine and threonine between TGY and JGY, especially the serine content of JGY in fresh leaves, which was 142.47 times greater than that in TGY. Despite TGY showing an increase of 326.46% in serine content after processing compared to that of JGY (156.72%), the final product of JGY still had a serine content 68.42 times greater than that of TGY (Fig. 4). The results indicated that high levels of serine and threonine at each stage are one of the potential reasons why TGY has a lower sweetness than JGY.

With the exception of the FL and WT stages, L-valine, derived from the pyruvate pathway, was the most significantly different metabolite between the two varieties, with extremely high levels at all stages of TGY. L-valine continuously accumulated during the manufacturing process, reaching its peak in the BF stage (Fig. 4). This accumulation of L-valine may be a mechanism in tea leaves for coping with various stress conditions through its use as a signaling component or metabolic prerequisite, which is consistent with the trend of increasing L-valine content in TGY oolong tea during the manufacturing process (Li et al., 2023). Notably, the L-valine in the fresh leaves of JGY was 118.04 times greater than that in the fresh leaves of TGY, reaching as high as 903.82 times that in the BF stage, after which it decreased after drying, although it remained 450.49 times higher in the tea product. The results showed that the significant difference in L-valine content in JGY fresh leaves became more pronounced after processing, which may be one of the potential reasons for the lower bitterness in TGY compared to that in JGY.

Glutamate serves as a signaling molecule for responding to external stress and is also the main contributing substance to the freshness of tea (Kaneko et al., 2006; Scharbert & Hofmann, 2005). Compared with JGY, TGY showed a higher accumulation of glutamate content at each stage (Fig. 4). The difference in glutamate content gradually became significant during processing (*p* < 0.05, VIP ≥ 1, FC ≥ 2 or ≤ 0.5, the same below) (Fig. 4). The peak values were reached in the FT stage for TGY and the WT stage for JGY, which may be related to drought and light stress caused by withering, as well as to slight mechanical damage (Shao et al., 2021; Zeng et al., 2019). Then, the glutamate content gradually decreased during the turnover process, indicating its involvement in plant metabolism. This result was basically consistent with the gradual decrease in glutamate after reaching its peak in the WT stage during the production of Zhangping Shuixian oolong tea (Wu et al., 2022). Specifically, during the manufacturing process (from FL to PT), the glutamate content decreased by 26.4% in TGY and by 64.9% in JGY. At the same time, the glutamate content in fresh leaves of TGY was 3.35 times greater than that in fresh leaves of JGY and 7.01 times greater than that in the tea products (Fig. 4). This indicated significant differences between TGY and JGY in terms of glutamate content in fresh leaves and an overall reduction during the manufacturing process, which may be one of the potential reasons for the greater umami of TGY compared to that of JGY.

In the metabolic pathway of tea, glutamate plays an important role as a common precursor for the synthesis of various protein amino acids (glutamine) and nonprotein amino acids (γ-aminobutyric acid and theanine). First, theanine, as one of the most representative nonprotein amino acids, plays a role in enhancing the umami taste of tea soup and offsetting its astringency and bitterness (Feng et al., 2014). It has a very low threshold, with an extraction rate in tea soup that can reach 80%. The theanine content in TGY was significantly greater than that in JGY, and significant differences were detected at each processing stage (Fig. 4). The theanine content in fresh leaves of TGY was 9.36 times greater than that in fresh leaves of JGY, which was 6 times greater than that in each processing stage. The results indicated that the accumulation of theanine was influenced more strongly by varietal factors than by external environmental factors, which is consistent with the findings of Yu and Yang (2020). Therefore, compared with those in JGY, the overall increase in theanine content in the fresh leaves and processing stages of TGY may be one of the potential reasons for its prominent umami.

Glutamine is the hub for regulating downstream alkaloids (Fig. 4). In tea plants, purine alkaloids are mainly present, among which caffeine, theobromine, and theophylline are important purine alkaloids. The complex formed by caffeine and tea polyphenols through hydrogen bonding (which has an umami taste), theobromine (an important precursor of caffeine), and theophylline (an isomer of theobromine) are all important bitter substances in tea. As shown in Fig. 4, there were no significant differences in the levels of these three substances between TGY and JGY (not meeting *p* < 0.05, VIP ≥ 1, FC ≥ 2 or ≤ 0.5). However, during the processing of TGY, except during the WT stage, their levels were greater than those in the processing of JGY at each stage, and the change trends were similar. Yu et al. (2014) conducted a depletion experiment on reconstituted tea soup and found that the removal of caffeine not only significantly reduced bitterness but also significantly reduced freshness. In addition, theaflavins are also very important for the convergence, freshness, and concentration of tea soup. Although there was no significant difference between TGY and JGY, their levels were also greater during the manufacturing process of TGY. After the FT stage, these substances began to increase significantly, reaching a peak at the TT stage, and then gradually decreased (Fig. 4). This may be because the turnover process destroys the tea leaf tissue structure, increases the permeability of the cell membranes, leads to the contact of enzymes with substrates, and induces oxidation reactions. With prolonged "Tanqing" and high-temperature treatment, enzyme activity is disrupted, and theaflavins gradually decrease. The results indicated that the overall contents of caffeine, theobromine, theophylline, and theaflavins in TGY are relatively high, which may be one of the potential reasons for the prominent fresh taste of TGY.

Flavonoid compounds produced by phenylalanine metabolism are important bitter taste substances in tea. Among them, catechins, as the main flavonoid compounds, have a significant impact on the flavor quality of tea leaves. As shown in Fig. 4, the contents of catechin and catechin gallate in each stage of JGY were significantly greater than those in TGY. Specifically, the average content of catechins was approximately 15 times greater in each stage. During the manufacturing process stages, their content continuously decreased, possibly due to enzymatic oxidation reactions. However, the contents of catechin and catechin gallate in the final tea product of JGY still showed significant differences of 15.11 times and 10.09 times greater than those in the TGY, respectively (Fig. 4). The results indicated that the differences in catechin content due to the variety and the manufacturing process are among the potential reasons for the weaker bitterness index of TGY compared to that of JGY.

Furthermore, based on the threshold and content of flavonoid glycosides (Guo et al., 2023), quercetin-3-O-galactoside (hyperin) and quercetin-3-O-rutinoside (rutin) are substances with small thresholds and large differences in content. Both flavonoid glycosides decreased during the manufacturing process (Fig. 4), but the content of the former was significantly greater than that of the latter in each stage of TGY, while the opposite trend was observed for JGY. Interestingly, during the manufacturing process stages, the rates of decrease of hyperin and rutin in TGY and JGY were basically the same. This indicated that variety may have a greater impact on hyperin and rutin than the manufacturing process does and that these two factors may be important underlying factors for the bitter taste of TGY and JGY.

Soluble sugars contribute to the sweetness of tea. The contents of six sugars, namely, galactose, glucose, fructose, erythrose, mannose, and rhamnose, in TGY were relatively low at all stages, among which galactose was the most significantly downregulated soluble sugar and erythrose was unique to JGY (Fig. 4). These results indicated that these soluble sugars may be one of the reasons for the weaker sweetness of TGY compared to that of JGY.

**Fig. 5:**
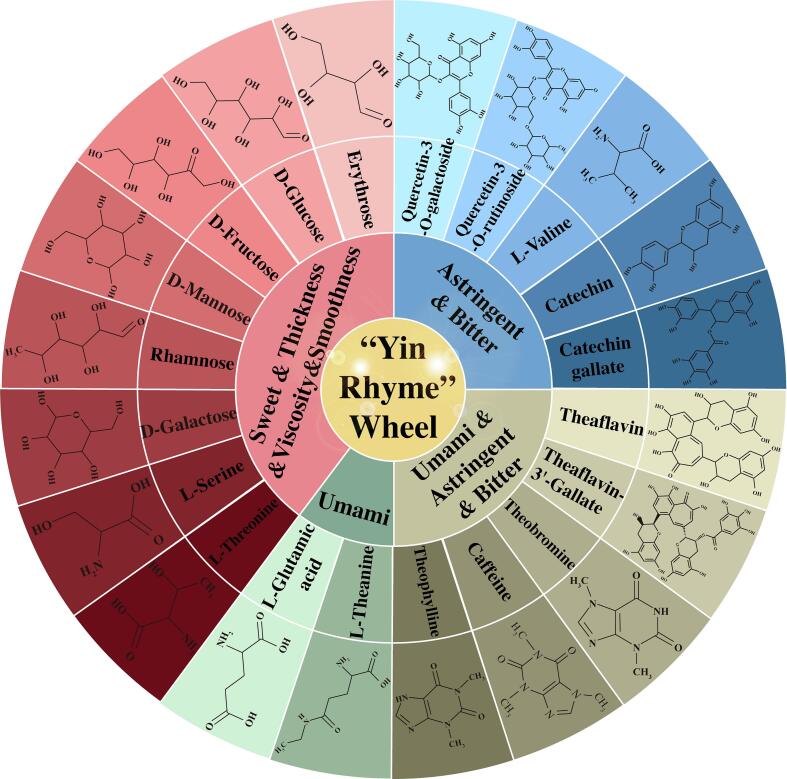
Summary of the substances that may be correlated result in the formation of the “Yin Rhyme” flavor in Tieguanyin oolong tea.

Overall, the common and significantly different substances identified during the stages of the manufacturing process of TGY and JGY were quite similar, indicating that the main flavor substances in the manufacturing process of the two were similar, but differences in their contents led to different taste profiles. Compared to those in JGY, the overall dynamic content of bitter taste characteristic components and sweet taste characteristic components in TGY was relatively low, while the overall dynamic content of fresh taste characteristic components was relatively high, contributing to the formation of the “Yin Rhyme” flavor. In previous research, we have also analyzed the key volatile substances linalool in TGY from the perspective of volatile substances compared to JGY (Zheng et al., 2022). Therefore, the high content of linalool and key nonvolatile substances showed in Fig. 5 will comprehensively coordinate the formation of “Yin Rhyme” of TGY.

## 4 Conclusion

This study conducted a comparative analysis, using TGY as the experimental material and comparing it with JGY as a reference material. Through a combination of sensory evaluation, electronic tongue (ET), and widely-targeted metabolomics approaches, we scrutinized the flavor attributes and nonvolatile taste components of JGY and TGY tea products, as well as the changes that occurred during processing. The results of sensory evaluation and electronic tongue analysis of the tea products both indicated that the taste of TGY tea products was more mellow and refreshing, with a strong “Yin Rhyme” flavor, while that of JGY was sweeter and milder. Widely-targeted metabolomics of the tea products revealed a total of 1317 nonvolatile metabolites, among which the content of sweet substances such as lactose, l-serine, and L-threonine, as well as that of bitter substances such as valine and catechin, was lower in TGY; conversely, the content of umami substances such as L-glutamic acid and L-theanine was greater, potentially contributing to the “Yin Rhyme” flavor characteristics of TGY. Despite similar caffeine levels between the two varieties, the complex formed by caffeine and theaflavins had a fresh and refreshing taste, enhancing its overall taste profile. The widely-targeted metabolomics results of each stage of the manufacturing process of oolong tea revealed significant differences in the content of 21 substances, including lactose, L-glutamic acid, L-theanine, valine, and catechin, in the fresh leaves of the two varieties, as well as different rates of increase and decrease during the manufacturing process, which were key to the formation of the “Yin Rhyme” flavor of TGY. Despite these achievements, the lack of targeted quantification of key flavor compounds, as well as research on the relationship between substance proportions and taste perception, hinders a better understanding of the formation of characteristic flavors in TGY. Further experiments on taste recombination in oolong tea processing should be conducted in the future. However, our study systematically analyzed the dynamic changes and differences in the taste of TGY and JGY oolong teas from the perspectives of sensory and metabolomics, providing a theoretical basis for the formation of the “Yin Rhyme” taste characteristics of TGY.

## CRediT authorship contribution statement

**Qiuming Li:** Writing – review & editing, Writing – original draft, Visualization, Formal analysis. **Qingcai Hu:** Writing – review & editing, Data curation. **Xiaoxi Ou:** Writing – review & editing. **Jihang He:** Writing – review & editing. **Xinru Yu:** Writing – review & editing. **Yunzhi Hao:** Writing – review & editing. **Yucheng Zheng:** Writing – review & editing, Project administration, Methodology, Data curation. **Yun Sun:** Writing – review & editing, Resources, Project administration, Funding acquisition.

## Declaration of competing interest

The authors declare that they have no known competing financial interests or personal relationships that could have appeared to influence the work reported in this paper.

## Data Availability

Data will be made available on request.
